# Synthesis and characterization of a Cu(ii) coordination-containing TAM radical as a nitroxyl probe†

**DOI:** 10.1039/d1ra07511j

**Published:** 2022-05-27

**Authors:** Wenbo Liu, Ouyang Tao, Li Chen, Yun Ling, Ming Zeng, Hongguang Jin, Dengzhao Jiang

**Affiliations:** School of Pharmacy and Life Sciences, Jiujiang University Jiujiang 332000 China 6090058@jju.edu.cn; School of Public Health, TianJin Medical University China

## Abstract

Nitroxyl (HNO) has been identified as an important signaling molecule in biological systems, and it plays critical roles in many physiological processes. However, its detection remains challenging because of the limited sensitivity and/or specificity of existing detection methods. Low-frequency electron paramagnetic resonance (EPR) spectroscopy and imaging, coupled with the use of exogenous paramagnetic probes, have been indispensable techniques for the *in vivo* measurement of various physiological parameters owing to their specificity, noninvasiveness and good depth of magnetic field penetration in animal tissues. However, the *in vivo* detection of HNO levels by EPR spectroscopy and imaging is limited due to the need for improved probes. We report the first “turn on-response” EPR probe for HNO utilizing a Cu(ii) coordination-containing TAM radical (denoted as Cu^II^[TD1]). Upon reaction with HNO, Cu^II^[TD1] shows a 16.1-fold turn-on in EPR signal with a low detection limit of 1.95 μM. Moreover, low-temperature EPR spectroscopic and ESI-MS studies showed that the sensing mechanism relies on the reduction of Cu(ii) by HNO. Lastly, Cu^II^[TD1] is selective for HNO over other reactive nitrogen and oxygen species except for some reductants (Cys and Asc). This new Cu(ii) coordination-containing TAM radical shows great potential for *in vivo* EPR HNO applications in the absence of reducing agents and provides insights into developing improved and targeted EPR HNO probes for biomedical applications.

## Abstract 2

Nitroxyl (HNO) has been identified as an important signaling molecule in biological systems, and it plays critical roles in many physiological processes.

## Graphical abstract

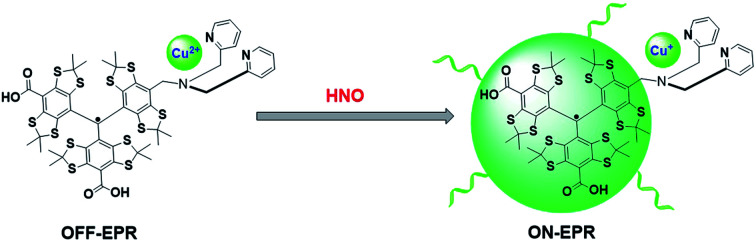

## 1. Introduction

Nitric oxide (NO) has received considerable attention due to its role as an active signal-inducing messenger biomolecule in immune systems.^1^ Nitroxyl (HNO) is the one-electron reduced and protonated product of nitric oxide (NO), which is a well-known signaling molecule in many physiological processes. Despite their very close structural similarity, it has recently been found that HNO shows different biological and chemical properties.^2–5^ HNO has biological activity. It has been theorized to be a potent cytotoxic agent that consumes cellular glutathione, causes double-stranded breaks in DNA,^6^ and elicitates smooth muscle relaxation.^7^ Recent studies on the biological role of HNO divulge that this species also has the ability to increase the cardiac output through the decrement of venous resistance, and as a result, HNO has been recognized to serve as a potential therapeutic agent for the treatment of myocardial ischemia–reperfusion injury^4^ and cardiovascular disorders.^8^ Although some progress regarding the biological chemistry of HNO has been achieved, the physiological and pathological effects of HNO still remain largely undiscovered. Thus, the sensitive and reliable detection of HNO in *in vitro* and *in vivo* systems is of paramount importance to understand its roles in normal physiology and disease.

Traditional methodologies for the detection of HNO are based on the analytical techniques of mass spectrometry,^9^ electrochemical analysis,^10^ high-performance liquid chromatography (HPLC),^11^ and the colorimetric method.^12^ Over the past decades, fluorescence methods have been widely used for HNO detection.^13–16^ Several fluorescent probes based on Cu(ii) complexes with a tripodal dipicolylamine (DPA)-appended receptor have been recently developed for the detection of biological HNO.^17–19^ However, these methods are mostly limited to *in vitro* or *ex vivo* detection due to their invasiveness and/or insufficient light penetration into tissues.

In recent years, great progress in low frequency electron paramagnetic resonance (EPR) instrumentation^20,21^ has been achieved, which allows for the *in vivo* measurement and mapping of different physiological parameters such as oxygen,^22^ redox status,^23,24^ pH^25^ and reactive oxygen species^26^ in isolated tissues and living animals. However, the full potential of this technique is still far from being realized due in part to the lack of spin probes that are stable, sensitive to HNO, have narrow linewidth (Δ*B*_pp_), and have target specificity to penetrate and localize within cells.

**Chart 1:**
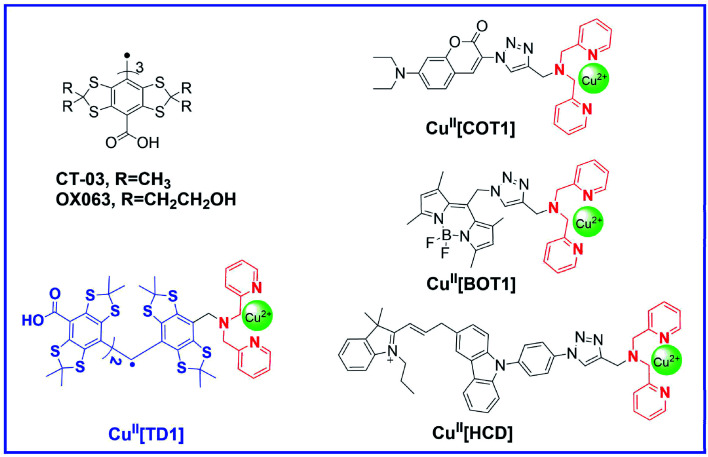
Molecular structures of HNO probes containing a tripodal dipicolylamine (DPA)-appended receptor.

Recently, tetrathiatriarylmethyl (TAM) radicals (OX063, CT-03, see Chart 1) have received wide attention as EPR probes owing to their high biostability and narrow singlet EPR signal at physiological pH, thereby providing more than 15-fold higher sensitivity and 200-fold improved time resolution for EPRI applications as compared to nitroxides.^27–31^ While the field of TAM probe development is still in its infancy, TAM radicals and their derivatives have been utilized to measure extracellular^32^ and intracellular^28,33^ oxygen levels, superoxide radical anions,^34^ pH,^35,36^ as well as redox status.^37,38^ Most recently, TAM radicals have shown great potential in proton electron double resonance imaging^39^ or PEDRI (also known as Overhauser magnetic resonance imaging or OMRI).^40^ In the present study, we propose a novel reaction-based “turn on-response” EPR probe for HNO utilizing a Cu(ii) coordination-containing TAM radical (denoted as Cu^II^[TD1], see Chart 1). We hypothesized that HNO could rapidly reduce Cu(ii) to Cu(i), resulting in the disappearance of the strong intramolecular spin exchange interaction, which would restore the EPR signal. This probe was found to provide excellent sensitivity and specificity for HNO detection. Therefore, our results represent the first example for the detection of HNO by the EPR method through a TAM-based probe.

## 2. Experimental section

### 2.1. EPR experiments and spectral simulation

EPR measurements were carried out on a Bruker EMX-plus X-band spectrometer at room temperature. The general instrumental settings were as follows: modulation frequency, 30–100 kHz; microwave power, 0.05–1 mW; modulation amplitude, 0.03–0.08 G. Measurements were performed in 50 μL capillary tubes. In addition, EPR measurements under anaerobic conditions were carried out using a gas-permeable Teflon tube (i.d. = 0.8 mm). Briefly, the experimental solution was transferred to the tube, which was then sealed at both ends. The sealed sample was placed inside a quartz EPR tube with open ends. Argon gas was bled into the EPR tube and the EPR spectrum was recorded after a 30 min equilibrium.

EPR spectral simulation was conducted by a home-made EPR simulation program (ROKI\EPR) developed by Antal Rockenbauer.^41^ The EPR experiments and spectral simulation technology were supported by Prof. Liu YP's group (Tianjin Key Laboratory on Technologies Enabling Development of Clinical Therapeutics and Diagnostics, School of Pharmacy, Tianjin Medical University, Tianjin 300070, P. R. China).

### 2.2. Stability studies towards biological oxidoreductants

Solutions of H_2_O_2_ (1 mM) in PBS buffer (pH 7.4, 50 mM) were used. ClO^−^ was generated from NaClO. NO_2_^−^ was generated from NaNO_2_. NO_3_^−^ was generated from NaNO_3_. Alkylperoxyl radicals (ROO˙) were generated by the thermolysis of 2,2′-azobis-2-methylpropanimidamide dihydrochloride (AAPH, 1 mM) at 37 °C. Peroxynitrite (ONOO^−^) was generated by the decomposition of SIN-1 (1 mM) at 37 °C in the presence of SOD (50 U mL^−1^). Hydroxyl radicals (HO˙) were continuously generated from the system consisting of Fe(iii)–NTA (0.1 mM) and H_2_O_2_ (1 mM). Superoxide (O_2_˙^−^) was generated using the xanthine (X)/xanthine oxidase (XO) system using XO (20 mU mL^−1^) and X (0.4 mM) in the presence of DTPA (0.1 mM). Nitric oxide (NO) was generated from a saturated NO aqueous solution (2 mM). Nitroxyl donor (HNO) was generated from sodium trioxodinitrate (Na_2_N_2_O_3_, Angeli's salt). Angeli's salt was prepared as described by King and Nagasawa, and was stored dry at −20 °C until needed.^42^ EPR spectra were recorded 30 min after mixing the TAM radical solution (20 μM) with various oxidoreductants. The effect of various reactive species on the TAM radical was expressed as a percentage of TAM radical remaining after exposure to the reactive species for 30 min, which was obtained by the double integral of the EPR signal. Each experiment was conducted three times.

## 3. Results and discussion

### 3.1. Synthesis

**Scheme 1:**
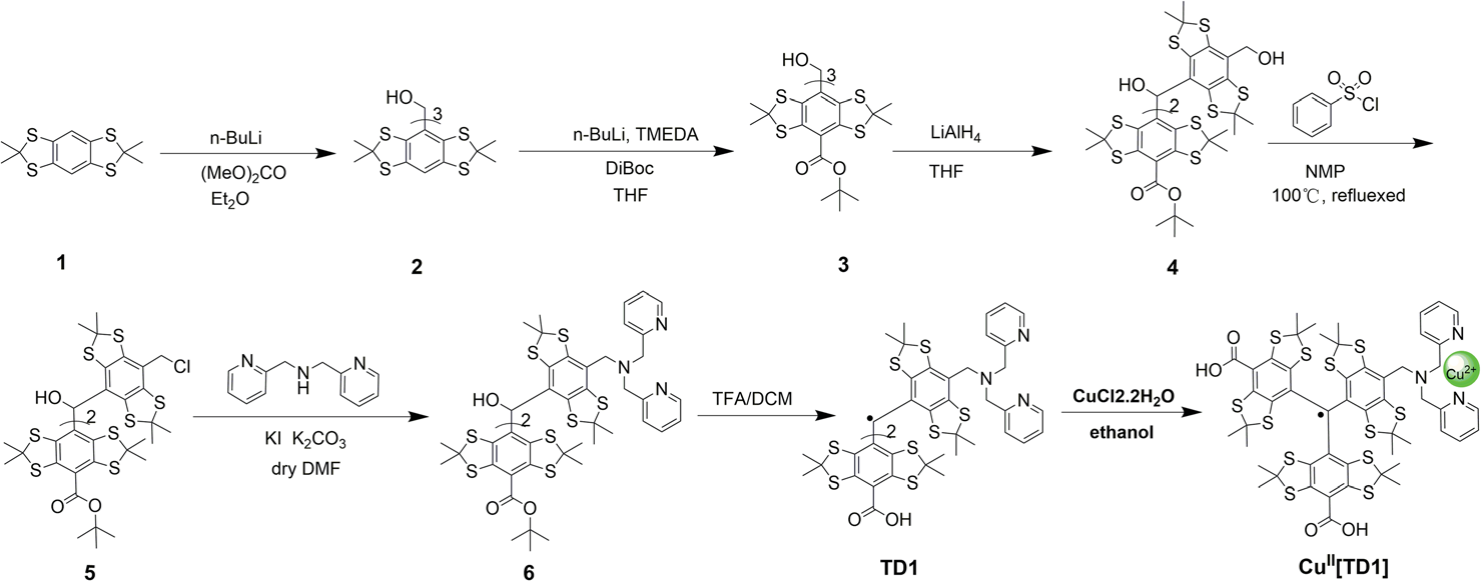
Synthesis of Cu^II^[TD1].

The synthesis of compound 2 proceeded as described in the literature with some minor changes (Scheme 1). Compound 1 was converted directly to the desired thioacetonide 2 by condensation with acetone in the presence of BF_3_·Et_2_O. Compound 1 was further converted into the intermediate thioacetonide 2 by heating under reflux with acetone. BF_3_·Et_2_O and chloroform were used as the catalyst and solvent, respectively, instead of HBF_4_ and toluene, which was recommended by the literature source.^43^ The treatment of arene 2 with 1 equiv. *n*-BuLi followed by 0.32 equiv. diethyl carbonate afforded trityl alcohol 3. Following the earlier method, trityl alcohol 3 was deprotonated with *tert*-BuLi and TMEDA, and the resultant anion was treated with excess di-*tert*-butyl dicarbonate (DIBOC) to afford the triester 4. The triester 4 was reduced partially by LiAlH_4_ to the corresponding benzyl alcohol 5. Then, the reaction of 5 with benzenesulfonyl chloride in the presence of N-methyl pyrrolidone led to the corresponding benzyl chloride 6. Subsequently, the SN_2_ reaction of 6 with di-(2-picolyl)amine afforded compound 7. Trityl TD1 was obtained by treatment of 7 with TFA/DCM. Finally, Cu^II^[TD1] was generated by the addition of CuCl_2_ to TD1.

### 3.2. EPR spectra of TD1

**Fig. 1:**
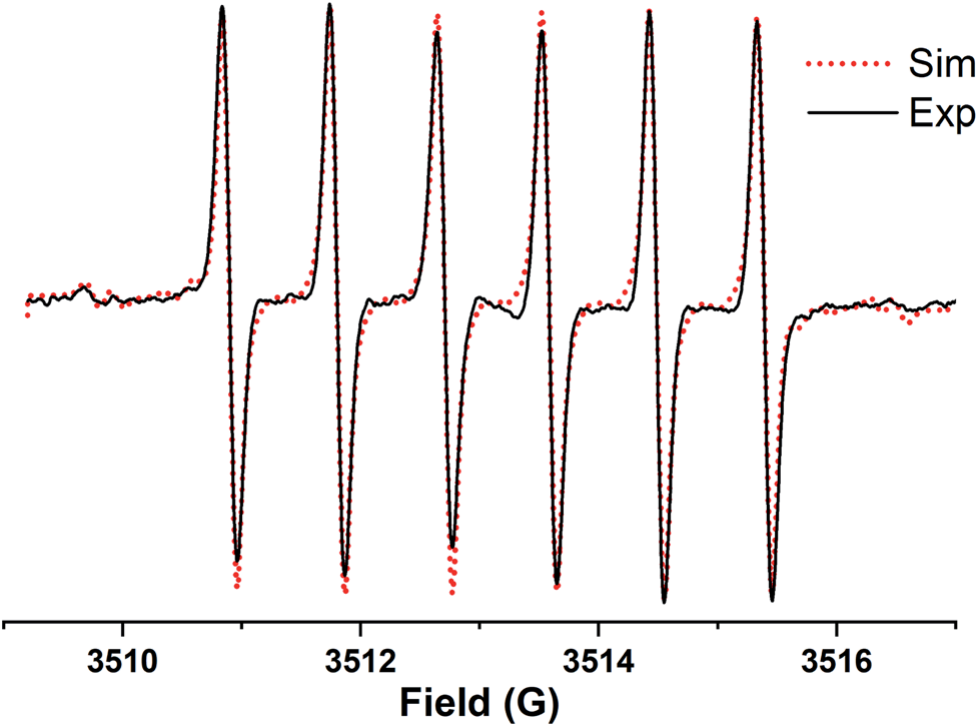
Experimental (black solid line) and simulated (red dotted line) EPR spectra of TD1 (10 μM) in PBS buffer (50 mM, pH 7.4) under anaerobic conditions. Spectral parameters: microwave power, 0.63 mW; time constant, 20.48 ms; conversion time, 80.0 ms; sweep time, 81.92 s; frequency, 9.3 GHz; modulation amplitude, 0.1 G; sweep width, 8 G; number of points, 1024. Dotted lines represent the calculated EPR spectra with peak-to-peak line widths, Δ*H*_L_ = 56 mG, and the following hfs constants: *a*_H_1__(CH_2_) = 2.69 G, *a*_H_2__(CH_2_) = 0.06 G and *a*_N_ = 0.91 G.

Fig. 1 shows the experimental and simulated EPR spectra of TD1 in PBS buffer under anaerobic conditions, which exhibit a symmetrical sextet signal with peak-to-peak line widths (56 mG) due to hyperfine splittings (hfs) from the two unequivalent protons on the methylene group and nitrogen nuclei on the DPA group. The simulation of the EPR spectra is in excellent agreement with the experimental spectra, which demonstrates the values of hfs for nitrogen and methylene hydrogen nuclei: *a*_H_1__(CH_2_) = 2.69 G, *a*_H_2__(CH_2_) = 0.06 G and *a*_N_ = 0.91 G.

### 3.3. Reaction of Cu^II^[TD1] with HNO

**Fig. 2:**
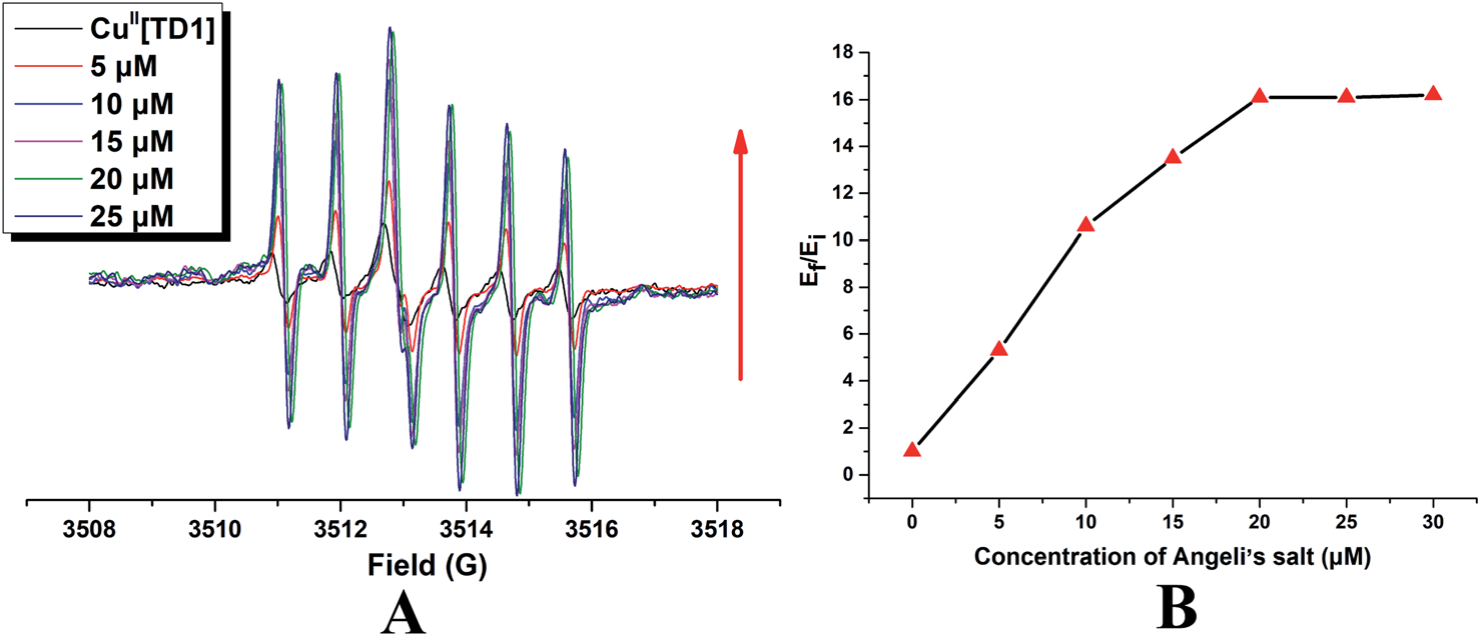
(A) Concentration-dependent EPR spectra obtained by incubating Cu^II^[TD1] (1 μM) with different amounts of Angeli's salt (Na_2_N_2_O_3_, a HNO donor) in PBS buffer (50 mM, pH 7.4) at room temperature. (B) Variation of EPR signal double integration (triangle) with different concentrations of Angeli's salt. *E*_f_/*E*_i_ represent the final (*E*_f_) over the initial (*E*_i_) double integral of the EPR signal.

**Fig. 3:**
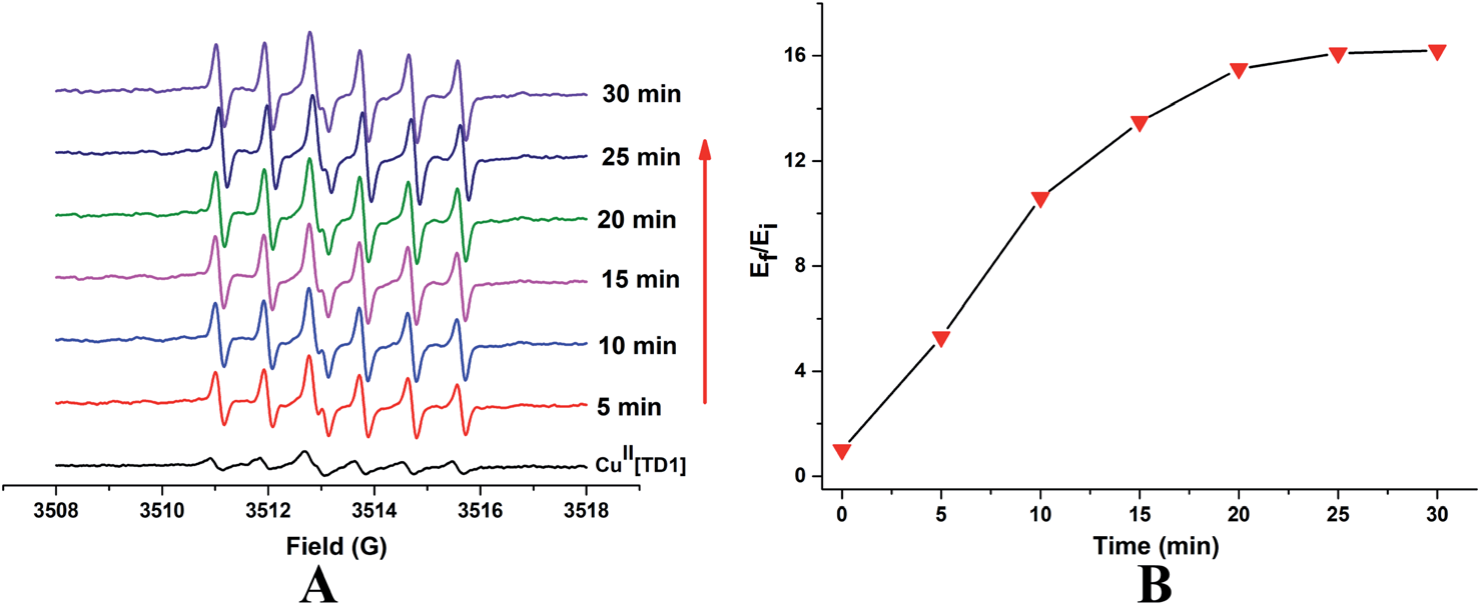
(A) Time-dependent EPR spectra obtained by incubating Cu^II^[TD1] (1 μM) with HNO (30 μM) in PBS buffer (50 mM, pH 7.4) at room temperature. (B) Variation of EPR signal double integration (triangle) with time in the presence of HNO. *E*_f_/*E*_i_ represents the final (*E*_f_) over the initial (*E*_i_) double integral of the EPR signal.

To check if Cu^II^[TD1] can function as a HNO detection probe, its reactivity with HNO was investigated by EPR. Compared with the EPR signal of TD1, Cu^II^[TD1] showed dramatic EPR signal quenching (17.8-fold) (Fig. S1†). Firstly, the concentration-dependent EPR signal of Cu^II^[TD1] towards HNO was investigated. The EPR signal intensity increased steadily with increase in the Angeli's salt (Na_2_N_2_O_3_, a HNO donor) concentration (Fig. 2) until it reached a plateau at 20 μM Na_2_N_2_O_3_, which corresponded to a 16.1-fold increase in the EPR signal intensity compared to that of blank [HNO]. This indicates that the complete reduction of Cu^II^[TD1] occurred with 20 μM Na_2_N_2_O_3_. In addition, the EPR signal response of Cu^II^[TD1] to HNO exhibited a linear relationship in the range of 0–20 μM HNO (Fig. S3†), and its detection limit was determined to be 1.95 μM (3*s*/*k*). Then, the time-dependent EPR signal of Cu^II^[TD1] was further obtained by incubating Cu^II^[TD1] (1 μM) with HNO (30 μM). The EPR intensity of Cu^II^[TD1] increased steadily with an increase in the reaction time until it reached a maxima at 25 min, which corresponded to a 16.1-fold increase (Fig. 3). Moreover, the reaction of Cu^II^[TD1] with HNO was corroborated by the ESI-MS spectra of the probe Cu^II^[TD1] and Cu^I^[TD1] (Fig. S15 and S16†). As shown in Fig. S15,† a major peak at *m*/*z* 1226.9742 (calcd. 1226.9797) corresponding to [TD1 + Cu(i)]^+^ was observed when 30 μM Na_2_N_2_O_3_ was added. By comparison, the probe Cu^II^[TD1] without HNO only exhibited a peak at *m*/*z* 1263.7606 (calcd. 1263.7657), which corresponded to [TD1 + Cu(ii) + Cl]^+^ (Fig. S16†). Low-temperature X-band EPR spectroscopy of Cu^II^[TD1] provided further evidence for the reduction of the paramagnetic Cu^II^[TD1] complex by HNO (Fig. S2†). In order to detect the EPR signal of Cu^II^, the EPR signal of Cu^II^[TD1] was measured under high mw power, high modulation amplitude and low temperature. As shown in Fig. S2,† the EPR spectra of Cu^II^[TD1] in EtOH exhibit a rhombic signal (LW = 470 G), which disappeared upon treatment with 30 equiv. HNO, as expected for the reduction of Cu(ii) to Cu(i). Thus, low-temperature EPR spectroscopic and ESI-MS studies showed that the sensing mechanism relies on the reduction of Cu(ii) by HNO. Taken together, Cu^II^[TD1] was sensitive to HNO and could be used to quantitate HNO.

### 3.4. Selectivity of Cu^II^[TD1] toward HNO

**Fig. 4:**
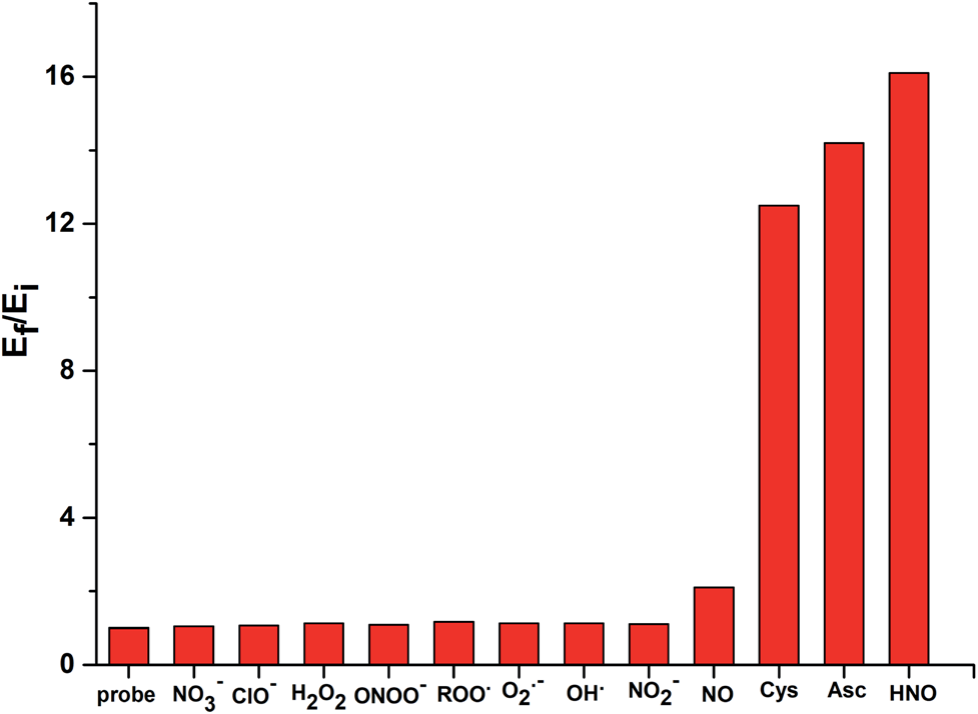
RPR responses of Cu^II^[TD1] (1 μM) to various 30 μM ROS and RNS species. Bars represent the final (*E*_f_) over the initial (*E*_i_) double integral of the EPR signal.

While Cu^II^[TD1] was highly sensitive to HNO, it was important to investigate its selectivity with various ROS and RNS species (Fig. 4). The probe exhibited a 16-fold increase upon interaction with HNO. However, a much weaker response was observed with other biologically relevant ROS and RNS species, including NO_3_^−^, ClO^−^, H_2_O_2_, HO˙, O_2_˙^−^, ONOO^−^, ROO˙, and NO_2_^−^. With NO, a 2.1-fold increase in EPR intensity was observed, and this relative lack of induced fluorescence response could be used to discriminate between NO and HNO. These results indicate that Cu^II^[TD1] shows high selectivity towards HNO over other ROS and RNS species. In addition, submillimolar cysteine and sodium ascorbate could also be used to restore the EPR signal intensity (12.5-fold and 14.2-fold, see Fig. S7 in the ESI†) because of the reduction of chelated Cu(ii)–DPA. To improve the stability and selectivity of TAM-based HNO probes, the Cu(ii)–DPA group was replaced with a Cu(ii)–azamacrocyclic group in the following work.^44^

## 4. Conclusions

In summary, a Cu(ii) coordination-containing TAM radical (Cu^II^[TD1]) was developed that acted as a turn on-response probe towards HNO for EPR detection. To the best of our knowledge, this is the first study showing the measurement of HNO by the EPR method using a TAM-based probe. However, the limitation of Cu^II^[TD1] is that reducing agents of biological origin such as cysteine and ascorbic acid can induce EPR signal turn-on, which limits its application *in vivo*. For future *in vivo* applications, the stability and selectivity of TAM-based HNO probes have been improved by replacing the Cu(ii)–DPA group with a Cu(ii)–azamacrocyclic group; the biocompatibility needs to be further improved possibly through PEGylated dendritic encapsulation;^45^ and lastly, the replacement of the methylene group with amido linkage would make the EPR signal of this probe much simpler and sharper. Overall, this new probe provides a powerful tool for noninvasively measuring HNO in a wide variety of chemical and biological systems, and provides important insights into the design of TAM-based HNO probes with improved properties.

## Conflicts of interest

There are no conflicts to declare.

## Supplementary Material

RA-012-D1RA07511J-s001
