# Supplementary material for: Synthesis and characterization of a Cu(ii) coordination-containing TAM radical as a nitroxyl probe
Source: RSC Adv. 2022 May 27;12(25):15980–5. doi: 10.1039/d1ra07511j (PMC9138401; doi:10.1039/d1ra07511j)
Supplement: RA-012-D1RA07511J-s001 [file RA-012-D1RA07511J-s001.pdf]

Electronic Supplementary Material (ESI) for RSC Advances.
This journal is © The Royal Society of Chemistry 2022

Supporting Information

Synthesis and Characterization of a Cu(II) Coordination–containing TAM Radical as Nitroxyl Probe

Wenbo Liu[1]*, Ouyang Tao[1], Li Chen[2], Yun Ling[1], Ming Zen[1], Hongguang Jin[1], Dengzhao Jiang[1]

[1] School of Pharmacy and Life Sciences, Jiujiang University, Jiujiang 332000, China

[2] School of Public Health, TianJin Medical University

Corresponding Author: bowenliu2008@163.com (Wenbo Liu)

Table of contents

1. Syntheticdetails S2-S3
2. EPR spectrum of TD1, Cu^{II}[TD1] in PBS and EPR spectra obtained by reaction of Cu^{II}[TD1] (1 μM) with HNO (30 μM) in PBS (50 mM, pH 7.4) after 30 min S4
3. Low-temperature X-band EPR spectra of **Cu^{II}[TD1](**50 μM) in EtOH (blue) and EPR spectra obtained by reaction of Cu^{II}[TD1] (50 μM) with HNO (1.5 mM) in EtOH (50 mM, pH 7.4) after 30 min (red). S4
4. Linear responses of **Cu^{II}[TD1]** for HNO S5
5. HPLC chromatograms of Compound **6** S5
6. HPLC chromatograms of TD1 S6
7. HPLC chromatograms of Cu^{II}[TD1] S6
8. EPR responses of Cu^{II}[TD1] in submillimolar cysteine and sodium ascorbate S7
9. ^{1}H NMR spectrum of compound **2** S7
10. ^{1}H NMR spectrum of compound **3** S8
11. ^{1}H NMR spectrum of compound **4** S8
12. ^{1}H NMR spectrum of compound **5** S9
13. ^{1}H NMR spectrum of compound **6** S9
14. Mass spectra of Compound **6** S10
15. Mass spectra of **TD1** S10
16. Mass spectra of **Cu^{II}[TD1]** S11
17. Mass spectra of **Cu^{I}[TD1]** S11

Synthetic details

Scheme 1 Synthesis of **Cu^{II}[TD1]**.

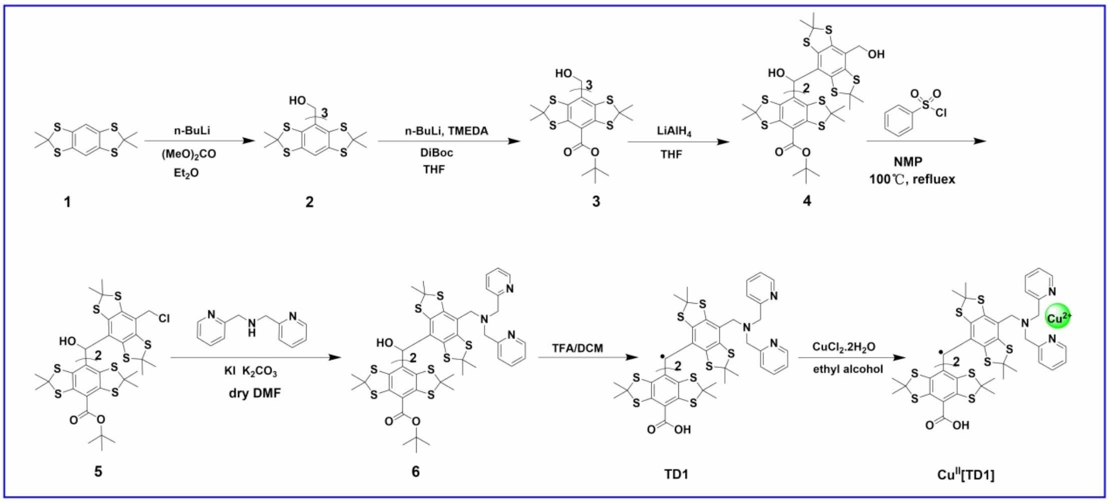

Compound 2: To a stirred solution of **1** (20 g, 69.8 mmol) in dry ether (600 mL), 2.5 M n-BuLi in hexanes (33.5 mL, 83.8 mmol) was added dropwise at room temperature Under Ar, and a white precipitate was observed. The mixture was stirred at room temperature for 3 h, dimethyl carbonate (2.0 mL, 23.3 mmol) was injected at a rate of 0.2 mL/h via a syringe pump. Then the color of the reaction changed from off-white to green. After 10 h, saturated aqueous NaH_2PO_4 (40 mL) was added and extracted with EA (200 mL x 2). The aqueous layer was extracted with DCM (70 mL x 3) and the combined organic layers were washed with saturated NaCl and dried over Na_2SO_4. The solvent was removed under reduced pressure to give the crude product, which was washed with petroleum ether (50 mL x 3). The orange residue was recrystallized with EA to give an off-white powder (9.39 g, 10.6 mmol, yield: 80%). ^{1}H NMR (400 MHz, $CDCl_3$) δ 7.17 (s, 3H), 6.21 (s, 1H), 1.82 (s, 9H), 1.80 (s, 9H), 1.72 (s, 9H), 1.68 (s, 9H).

Compound 3: To the suspension of **2** (10 g, 11.29 mmol) and TMEDA (17.0 mL, 112.9 mmol) in THF(250 mL), 2.5 M n-BuLi in hexanes (45.2 mL, 112.9 mmol) was added over 30 min at -20°C under Ar. Then the mixture was stirred at room temperature for 4 h. The resulting brown pasty mixture was slowly injected into di-*tert*-butyl dicarbonate (123.2 g, 564.5 mmol) at -20°C under Ar. The resulting dark brown solution was allowed to reach room temperature gradually and stirred for 24 h. The reaction was quenched by the slow addition of methanol at - 20°C. The residue solution was concentrated under reduced pressure to give the crude product which was dissolved in 100 ml of DCM and washed with water (3 x 50 ml). The organic phase was dried over Na_2SO_4, filtrated, and evaporated under vacuum. The resulting residue was purified by column chromatography on silica gel using eluting with DCM/hexanes = 1: 6 to 1:1 as a yellow powder (6.6 g) with a yield of 50%. ^{1}H NMR (400 MHz, $CDCl_3$, δ): 6.73 (s, 1H), 1.77 (s, 9H), 1.74 (s, 9H), 1.66 – 1.65 (m, 45H).

Compound 4: To the solution of Compound 3 (3 g, 2.5 mmol) in dry THF(100 ml), 1 M $LiAlH_4$ in THF (5 ml,5.0 mmol) was added drop wise at -20°C Under Ar. The reaction was allowed to warm up to room temperature and the reaction was stirred for 10 min at RT. The excess $LiAlH_4$ was quenched with a few drops of 6% citric acid and the colour of the resulting mixtures turned from green to orange. The resulting solution was diluted with 50 ml of EA, and the organic phase

was washed with brine (2 x 50 mL). The organic phase was dried over Na_2SO_4, filtrated, and evaporated under vacuum to give the crude product which was purified by column chromatography eluted with DCM/hexanes = 2:1 to give the title product **4** as a yellow powder (1.1 g, 1.0 mmol, yield: 40%). ^{1}H NMR (400 MHz, $CDCl_3$): δ 6.69 (s, 1H), 4.72 (d, *J* = 12.5 Hz, 1H), 4.66 (d, *J* = 12.4 Hz, 1H), 1.94 (s, 1H), 1.79 (m, 15H), 1.75 – 1.69 (m, 9H), 1.64 (m, 30H).

Compound 5: To the suspension of **4** (0.8 g, 0.71 mmol) in N-Methyl pyrrolidone (1 ml), benzenesulfonyl chloride (0.9 mL, 7.9 mmol) was added under Ar. Then the mixture was refluxed for 30 min. The reaction was quenched with water, and extracted with DCM (20 mL x 2), the organic layers were washed with saturated NaCl and dried over Na_2SO_4, filtrated, and evaporated under vacuum to give the crude product which was purified by column chromatography eluted with DCM/hexanes = 1:4 to give the title product **5** as a yellow powder (0.667 g, 0.59 mmol, yield: 83%). ^{1}H NMR (400 MHz, $CDCl_3$): δ 6.69 (s, 1H), 4.72 – 4.55 (d, J = 2.4, 2H), 1.79 (m, 16H), 1.73 (d, J = 2.7 Hz, 8H), 1.69 – 1.59 (m, 30H)。

Compound 6: To the suspension of **5** (0.15 mg, 0.13 mmol) , anhydrous K_2CO_3 (179 mg, 1.3 mmol) and anhydrous KI (21.58 mg, 0.13 mmol) in dry DMF(3 mL), Di-(2-picolyl)amine (50 μL, 0.26 mmol) in dry DMF added dropwise under Ar. Then the mixture was refluxed for 4 h. The reaction was quenched with saturated aqueous NaH_2PO_4. The resulting solution was diluted with 100 ml of DCM, and the organic phase was washed with brine (2 x 50 mL). The organic phase was dried over Na_2SO_4, filtrated, and evaporated under vacuum to give the crude product which was purified by column chromatography eluted with DCM/MeOH = 100:2 to give the title product **6** as a yellow powder (0.13 g, 0.10 mmol, yield: 80%). ^{1}H NMR (400 MHz, $CDCl_3$): δ 8.51 (s, 2H), 7.65 (d, *J* = 6.7 Hz, 2H), 7.55 (d, *J* = 7.5 Hz, 2H), 7.15 (d, *J* = 5.1 Hz, 2H), 6.66 (s, 1H), 3.77 (m, 6H), 1.76 (s, 18H), 1.64 (s, 36H). Purity: 95% by HPLC (see Fig. S3). MS (ESI, $[M+H]^+$): 1296.1954 (measured), 1296.1958 (calculated) (see Fig. S11).

Compound TD1: Compound 6 (60 mg, 0.046 mmol) was dissolved in 2 ml of TFA and stirred for 1 h at RT. The solution was evaporated under vacuum, and the residue was dissolved in PBS (0.1 M, pH 7.4) and purified by column chromatography on reversed-phase C-18 using water followed by 0-50% acetonitrile in water as eluants to give the compound **TD1** as a green solid (20.2 mg, 0.017 mmol, 37%). Purity: 95% by HPLC (see Fig. S4). MS (ESI, $[M+H]^+$): 1167.0693 (measured), 1167.0679 (calculated) (see Fig. S12).

Compound Cu^{II}[TD1]: To a solution of **TD1** (10 mg, 0.85 μmol) in ethanol (0.5 mL) was added $CuCl_2.2H_2O$ (2.19 mg, 1.28 μmol) solution in ethanol (0.5 mL). The mixture was stirred for about 30 min until the starting material had been completely consumed as detected by TLC. 6 mL of Hexane was added to the mixture and the complex was precipitated from solution. The product was obtained by filter as green solid (9.45 mg, 0.76 μmol, 90%). Purity: 95% by HPLC (see Fig. S5). MS (ESI, $[M+H]^+$): 1263.7606 (measured), 1263.7657 (calculated) (see Fig. S13).

Supplementary spectra data

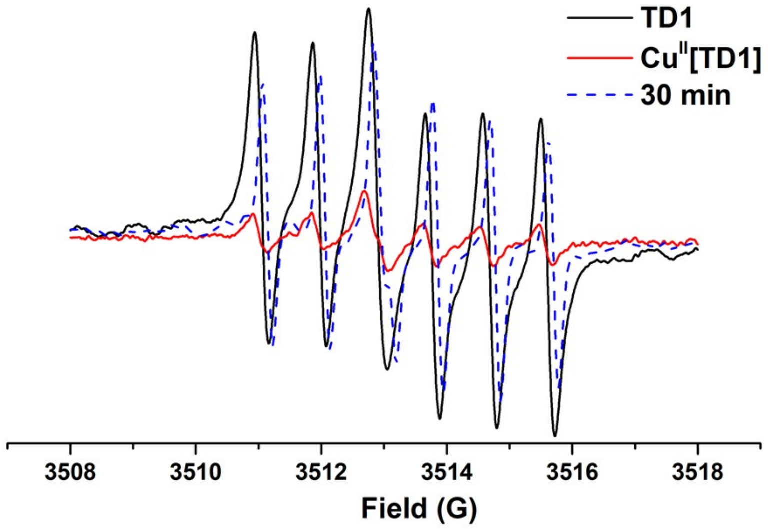

Fig. S1 EPR spectrum of TD1, Cu^{II}[TD1] in PBS and EPR spectra obtained by reaction of Cu^{II}[TD1] (1 μM) with HNO (30 μM) in PBS (50 mM, pH 7.4) after 30 min.

As shown in Fig. S1, to the spin quenching caused by strong intramolecular spin exchange interaction between the paramagnetic Cu(II) and trityl radical, Cu^{II}[TD1] showed a dramatic EPR signal quenching (17.8-fold). Upon incubation of HNO for 30 min (the response time), paramagnetic Cu(II) was reduced to diamagnetic Cu(I), which result in a relatively sharp sextet signal. The maximum EPR recovery signal can reach to 90% of TD1.

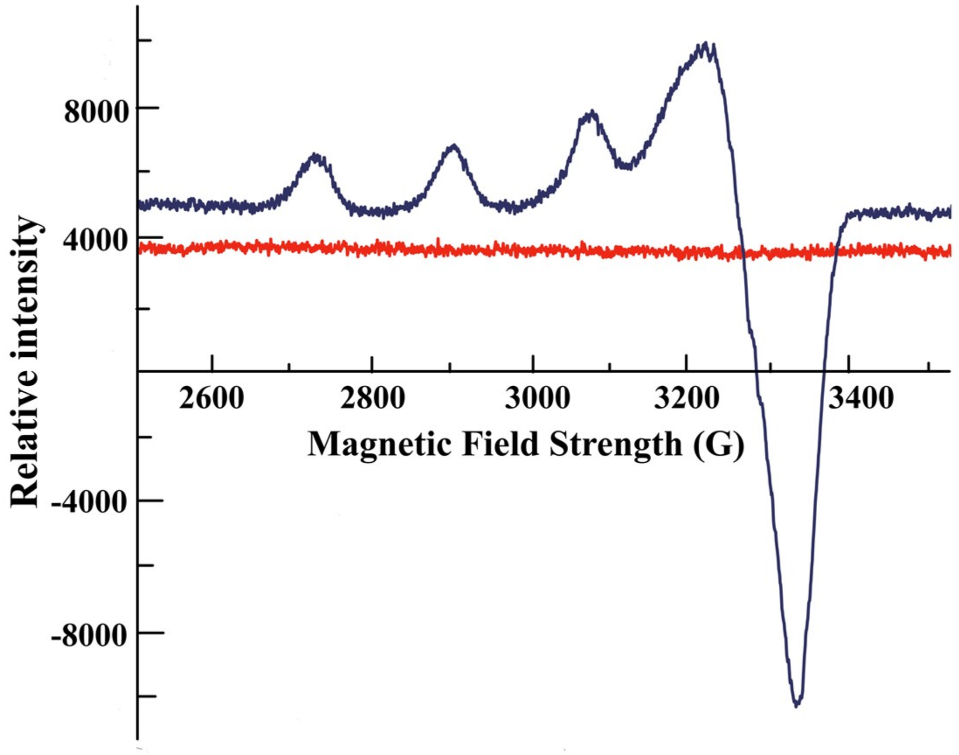

Fig.S2 Low-temperature X-band EPR spectra of **Cu^{II}[TD1]**(50 μM) in EtOH (blue) and EPR spectra obtained by reaction of Cu^{II}[TD1] (50 μM) with HNO (1.5 mM) in EtOH (50 mM, pH 7.4)

after 30 min (red). X-band EPR spectra were recorded on a Bruker EMX EPR spectrometer. Collection parameters: temperature, 77 K; modulation amplitude, 20 G; microwave power, 0.2 mW at 9.23 GHz.

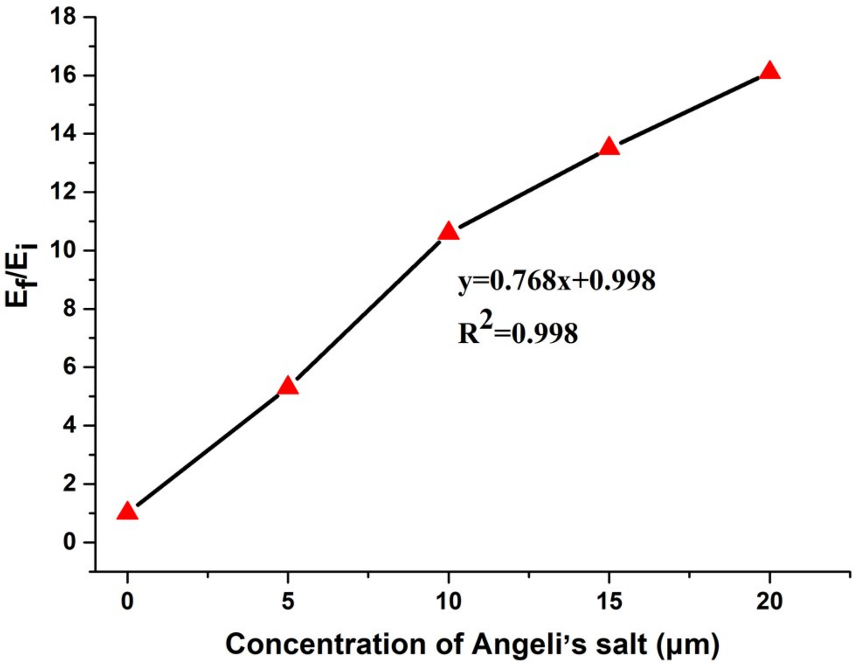

Fig.S3 Linear responses of **Cu^{II}[TD1]** for HNO. The detection limit of Cu^{II}[TD1] was determined to be 1.95 μM (3s/k). s is the standard deviation of measurements of blank simple for 10 times, k is the slope between intensity versus sample concentration.

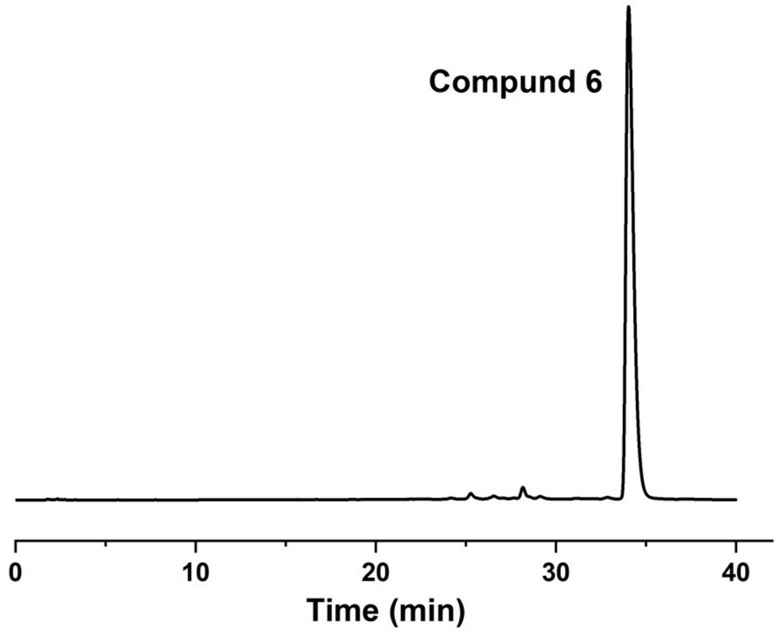

Fig. S4 HPLC chromatograms of Compound **6**.

The HPLC system (CoulArray System from ESA Analytical, Ltd. Chelmsford, MA) on a C18 Tosoh Bioscience ODS-80Tm column (250mm x 4.6mm, 5 micron) with refrigerated autosampler was used. ESA software was used for data collection and analysis. The mobile phase

consists of 20 mM ammonium acetate: acetonitrile = 40 : 60 (v : v) – 5 : 95 (v : v) at a flow rate of 1 ml/min which was filtered and degassed before use. Compound 6 was detected at 254 nm using a UV detector. The retention time is 34.03 min. The purity of Compound **6** was determined to be 95%.

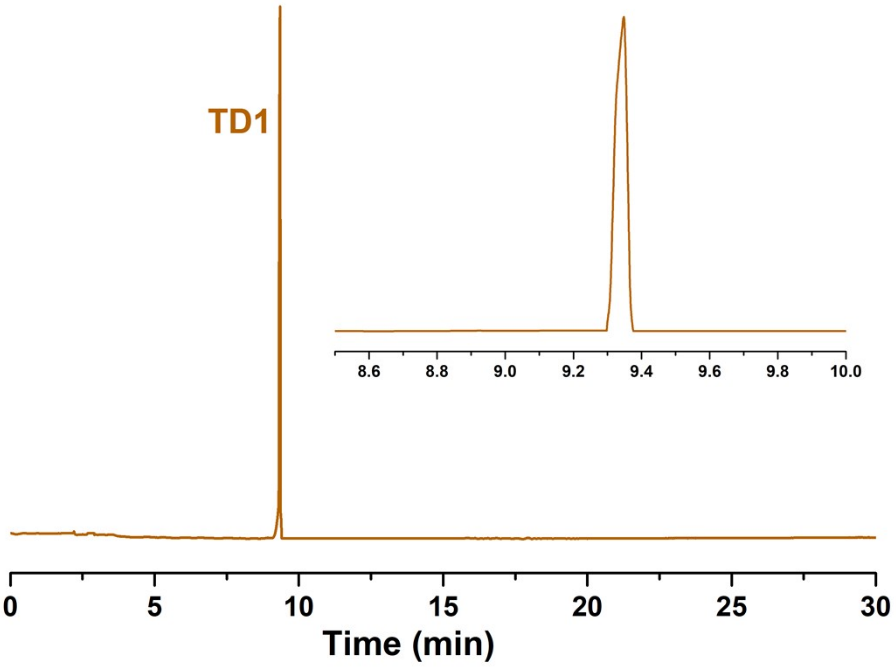

Fig. S5 HPLC chromatograms of TD1.

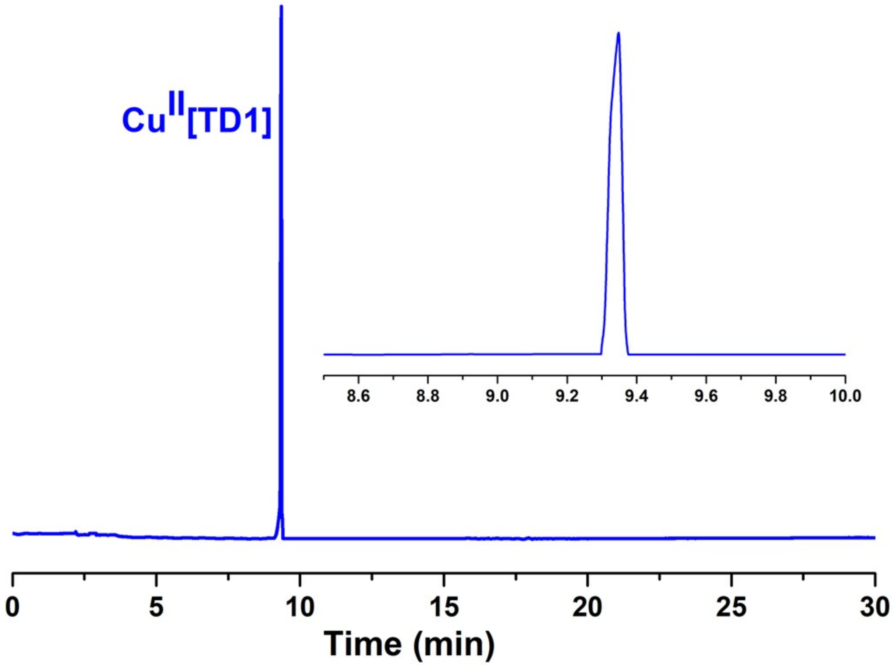

Fig. S6 HPLC chromatograms of Cu^{II}[TD1].

The HPLC system (CoulArray System from ESA Analytical, Ltd. Chelmsford, MA) on a C18 Tosoh Bioscience ODS-80Tm column (250mm x 4.6mm, 5 micron) with refrigerated autosampler was used. ESA software was used for data collection and analysis. The mobile phase consists of 20 mM ammonium acetate: acetonitrile = 30 : 70 (v : v) (pH 7.4) at a flow rate of 1

ml/min which was filtered and degassed before use. TD1 and Cu^{II}[TD1] were detected at 254 nm using a UV detector. The retention time is 9.32 min. The purity of TD1 and Cu^{II}[TD1] were determined to be 95%.

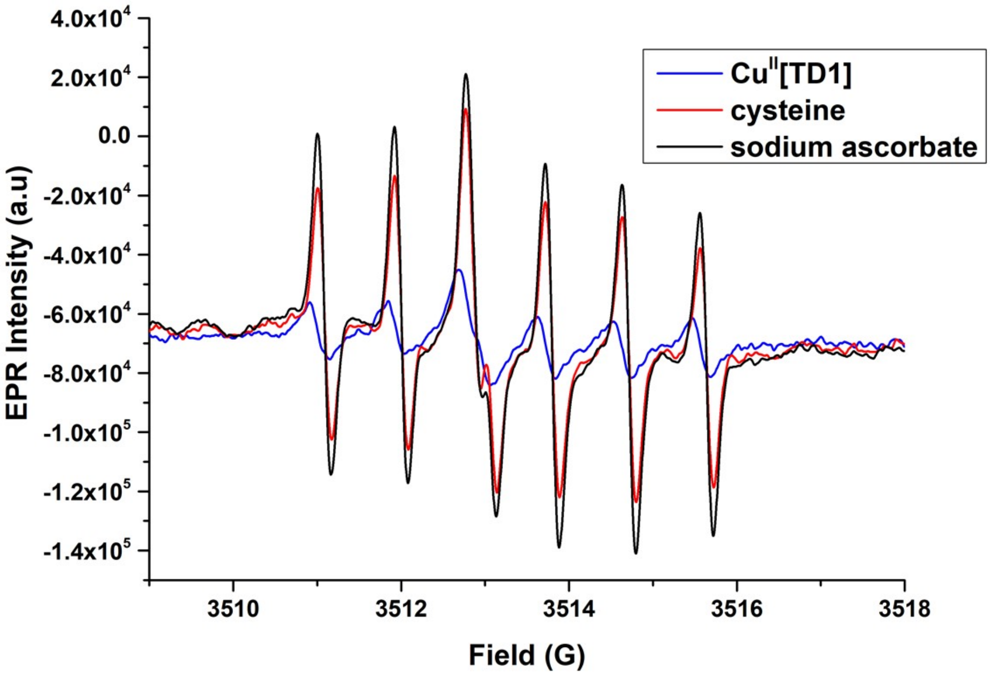

Fig. S7 EPR responses of Cu^{II}[TD1] in submillimolar cysteine and sodium ascorbate.

^{1}H NMR, ^{13}CNMR and ESI-MS spectra

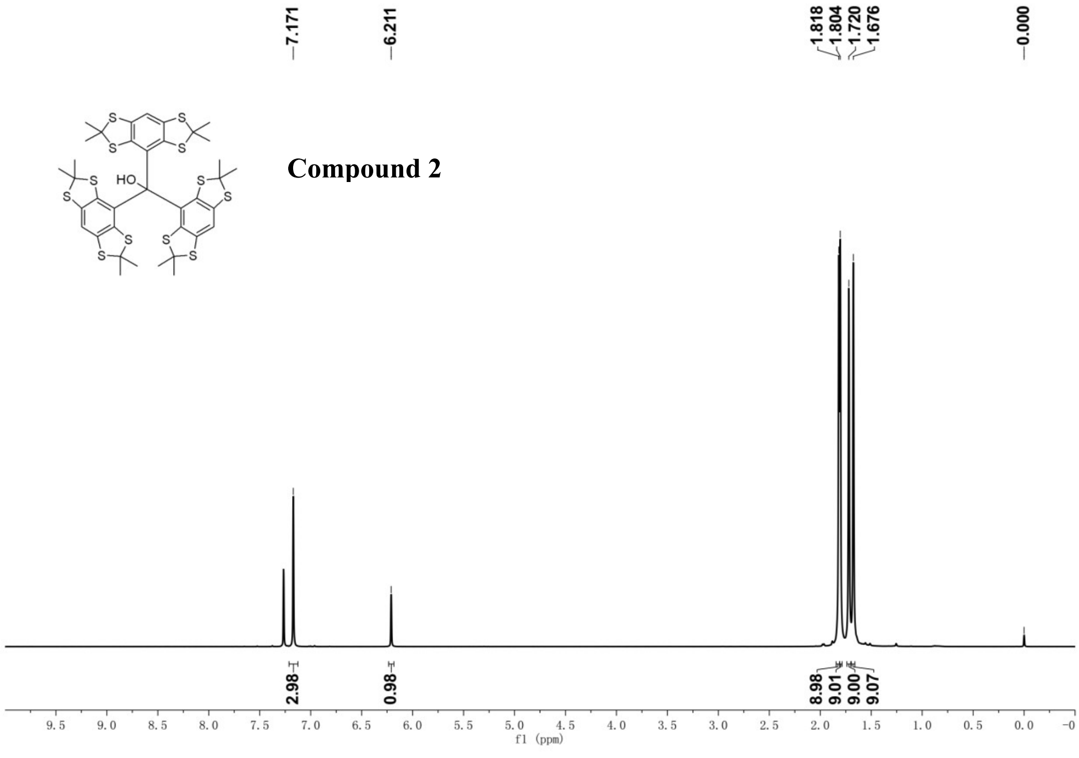

Fig. S8 ^{1}H NMR spectrum of compound **2** in $CDCl_3$.

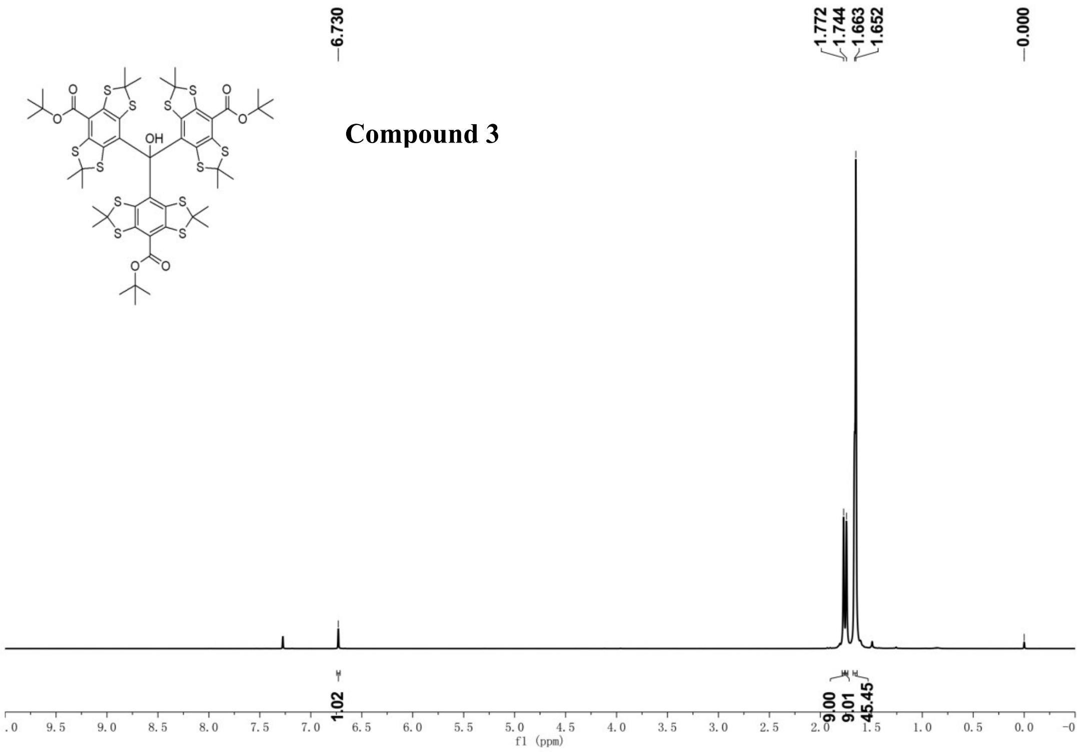

Fig. S9 ^{1}H NMR spectrum of compound **3** in $CDCl_3$.

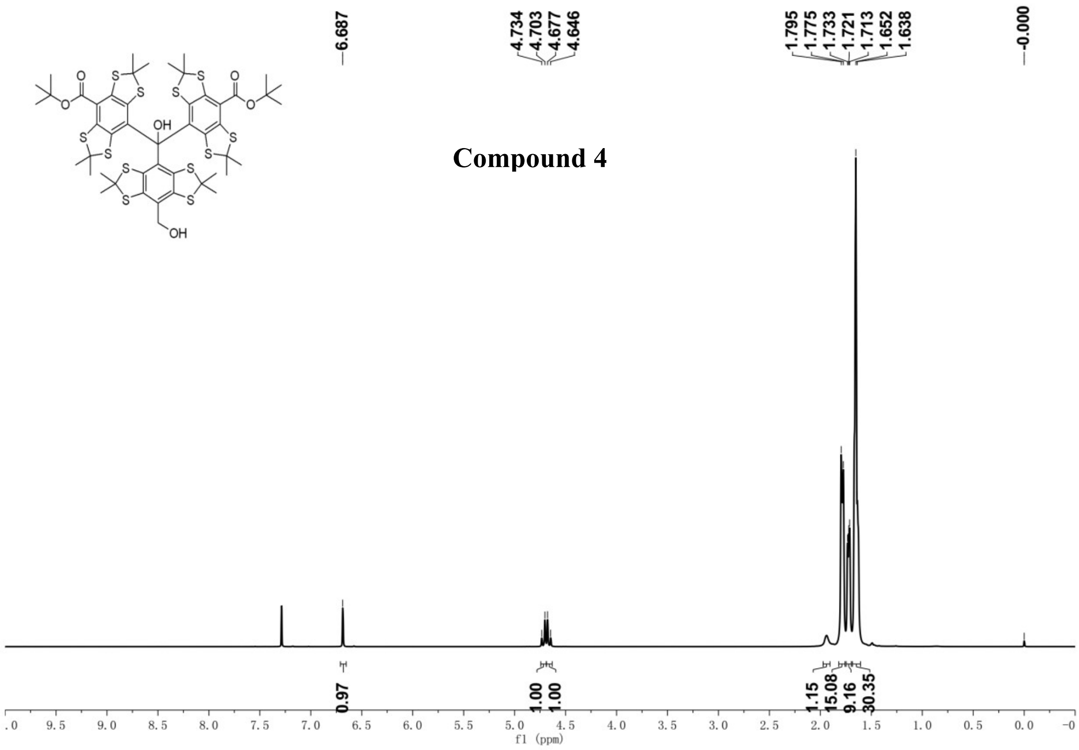

Fig. S10 ^{1}H NMR spectrum of compound **4** in $CDCl_3$.

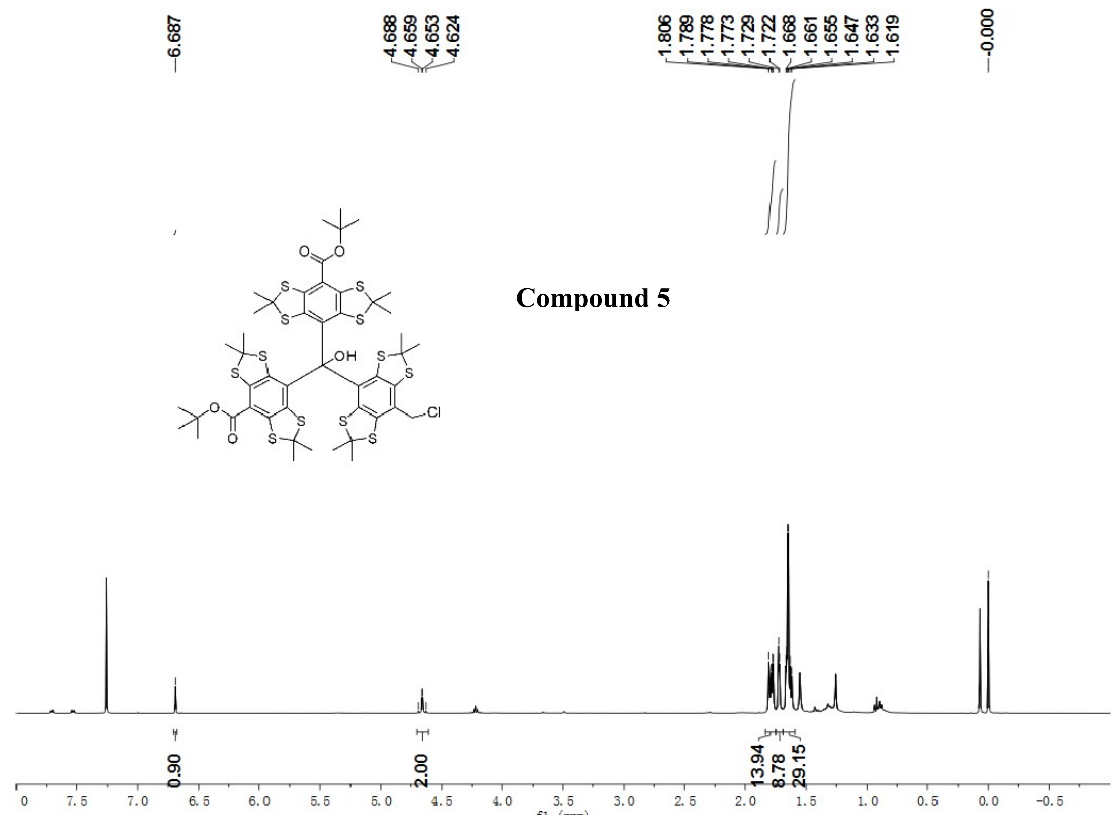

Fig. S11 ^{1}H NMR spectrum of compound **5** in $CDCl_3$.

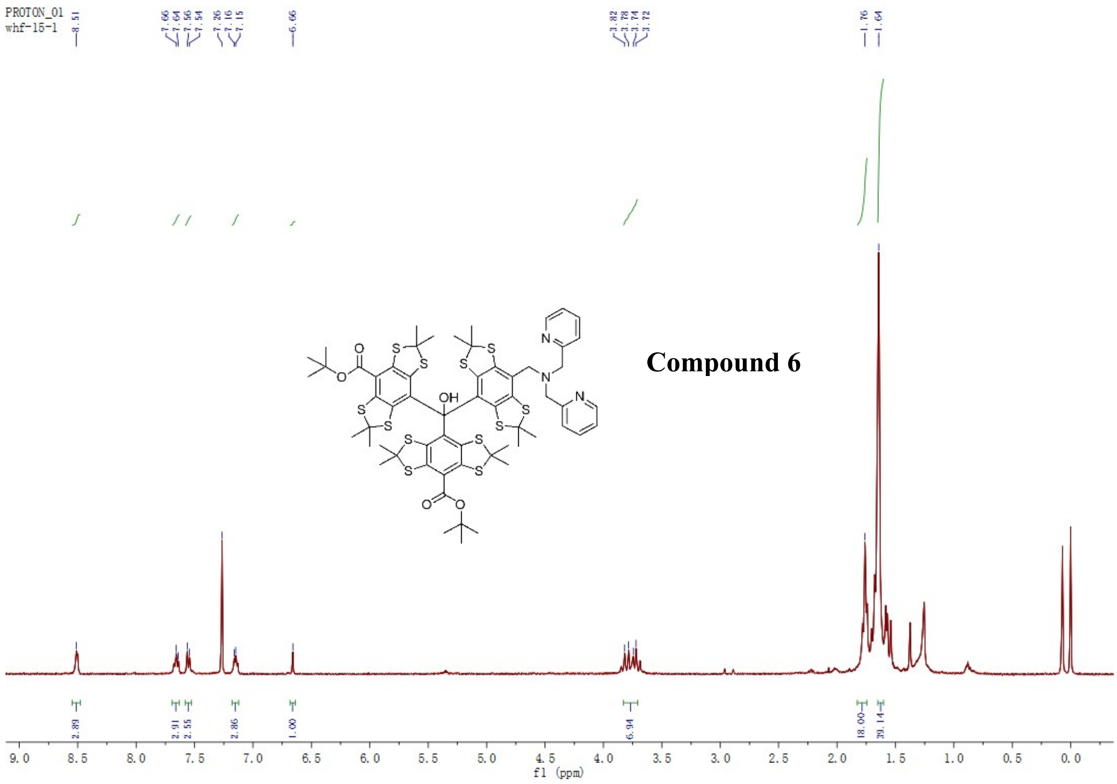

Fig. S12 ^{1}H NMR spectrum of compound **6** in $CDCl_3$.

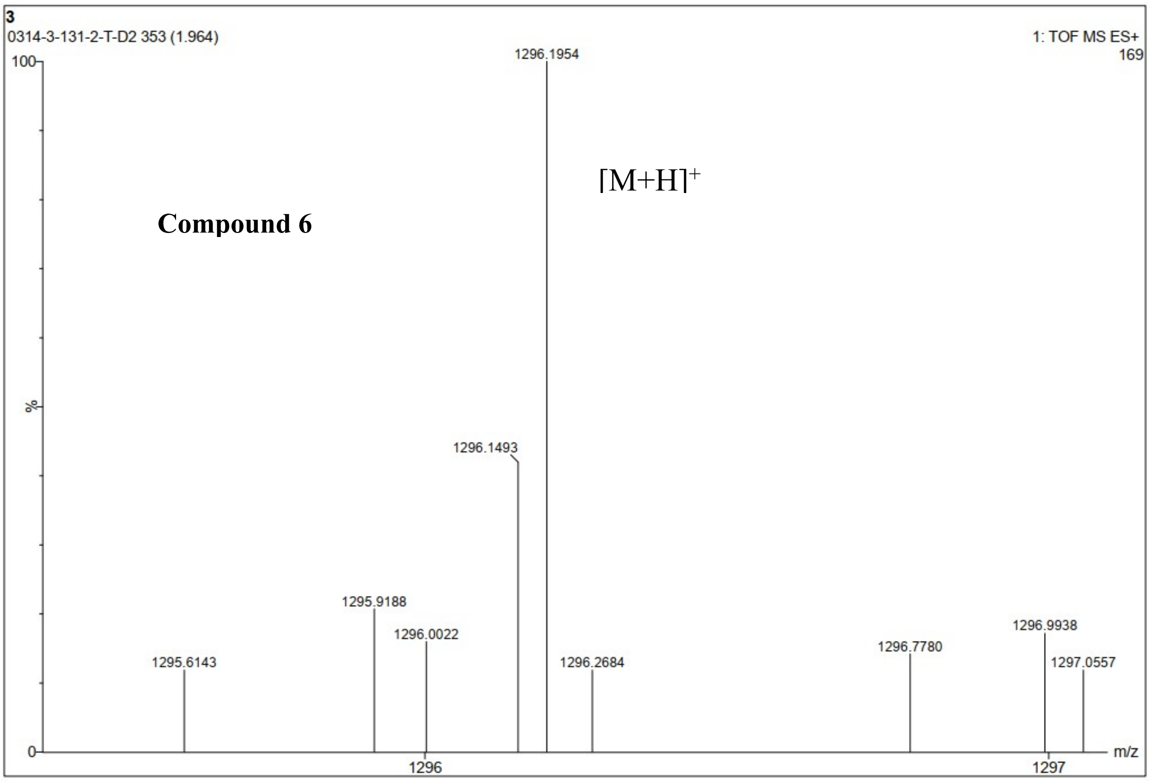

Fig. S13 Mass spectra of Compound **6**.

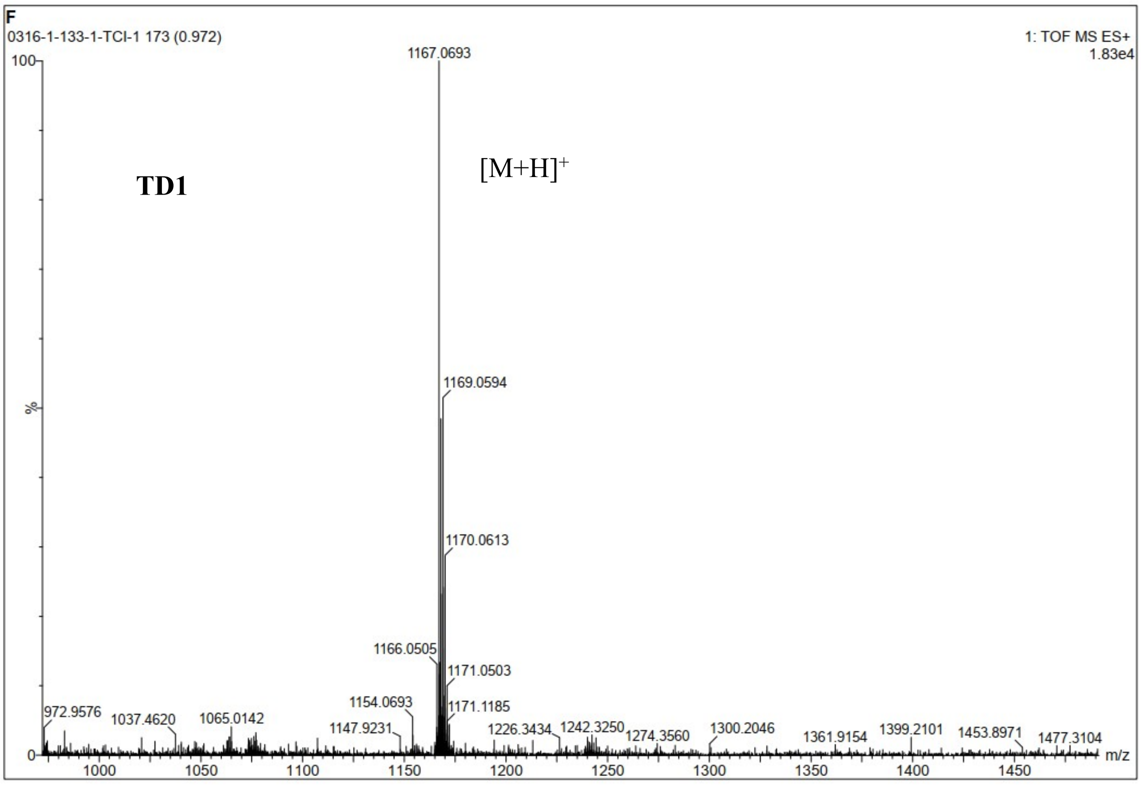

Fig. S14 Mass spectra of **TD1**.

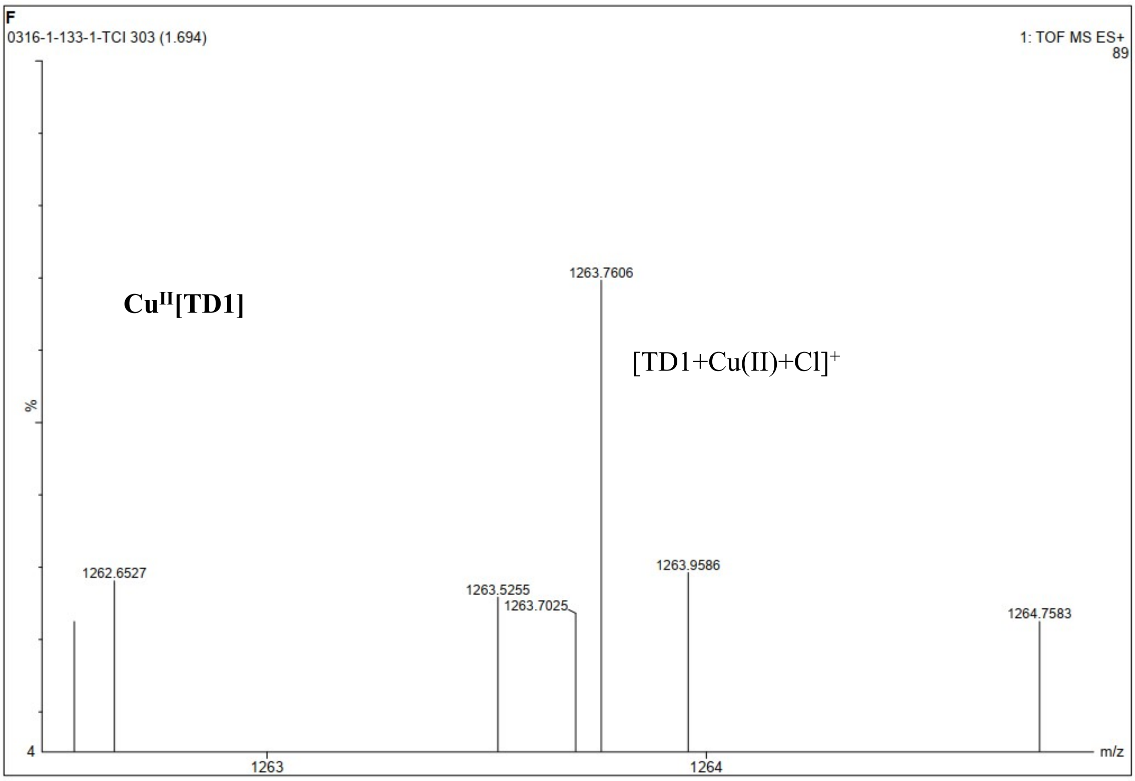

Fig. S15 Mass spectra of **Cu^{II}[TD1]**.

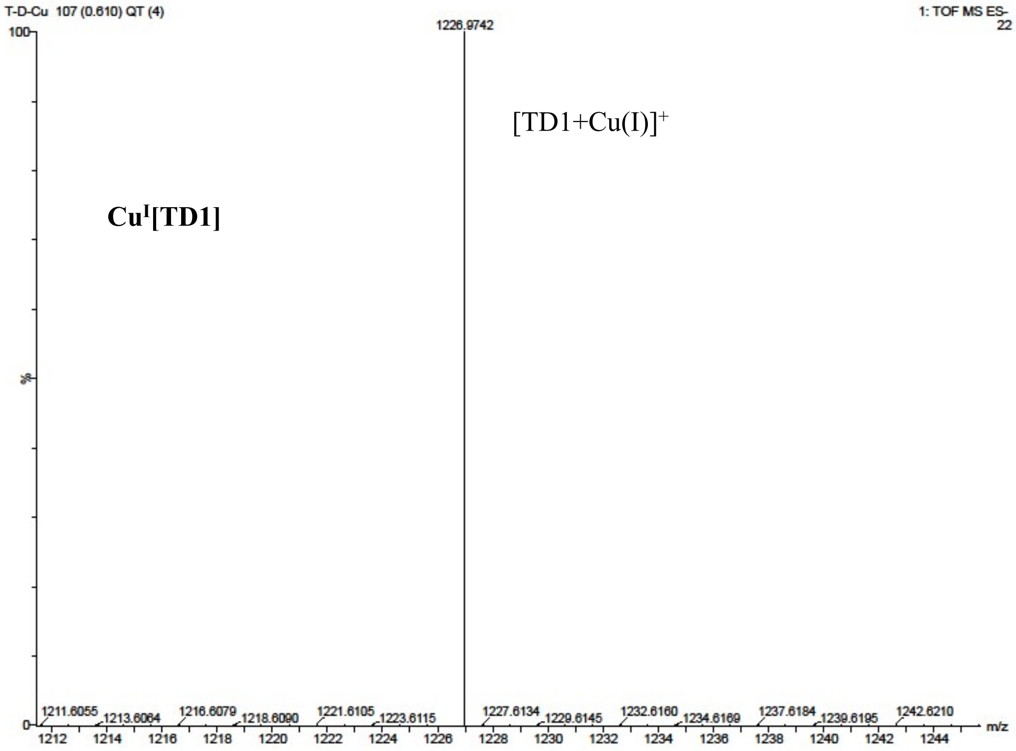

Fig. S16 Mass spectra of **Cu^{I}[TD1]**.
